# Differences in bacteria nanomotion profiles and neutrophil nanomotion during phagocytosis

**DOI:** 10.3389/fmicb.2023.1113353

**Published:** 2023-03-23

**Authors:** Svetlana Nikolaevna Pleskova, Ekaterina Vladimirovna Lazarenko, Nikolay Alexandrovich Bezrukov, Sergey Zenonovich Bobyk, Alexey Vladimirovich Boryakov, Ruslan Nikolaevich Kriukov

**Affiliations:** ^1^Laboratory of Scanning Probe Microscopy, Lobachevsky State University of Nizhny Novgorod, Nizhny Novgorod, Russia; ^2^Department of Nanotechnology and Biotechnology, R.E. Alekseev Nizhny Novgorod State Technical University, Nizhny Novgorod, Russia; ^3^Department of Semiconductors, Electronics and Nanoelectronics Physics, Lobachevsky State University of Nizhny Novgorod, Nizhny Novgorod, Russia

**Keywords:** nanomotion, bacteria, phagocytosis, atomic force microscopy, clinical isolates, oscillation, neutrophil granulocyte, frequency response

## Abstract

The main goal of this work is to highlight the connection between nanomotion and the metabolic activity of living cells. We therefore monitored the nanomotion of four different clinical strains of bacteria (prokaryotes) and the bacterial phagocytosis by neutrophil granulocytes (eukaryotes). All clinical strains of bacteria, regardless of their biochemical profile, showed pronounced fluctuations. Importantly, the nature of their nanomotions was different for the different strains. Flagellated bacteria (*Escherichia coli*, *Proteus mirabilis*) showed more pronounced movements than the non-flagellated forms (*Staphylococcus aureus*, *Klebsiella pneumoniae*). The unprimed neutrophil did not cause any difference in cantilever oscillations with control. However, in the process of phagocytosis of *S. aureus* (metabolically active state), a significant activation of neutrophil granulocytes was observed and cell nanomotions were maintained at a high level for up to 30 min of observation. These preliminary results indicate that nanomotion seems to be specific to different bacterial species and could be used to monitor, in a label free manner, basic cellular processes.

## 1. Introduction

Cell nanomotion (Venturelli et al., 2020) is a significant discovery, which could potentially lead to numerous biological and medical applications. Several scientific groups actively promote the atomic force microscope (AFM)-based method to detect bacterial nanomotion to determine the sensitivity and/or resistance of bacteria to antibiotics: the end of the nanomotion indicates the death of bacteria and the effectiveness of the drug (Longo et al., 2013; Kasas et al., 2015). This allows getting the most important clinical data on antibiotic resistance in minutes as opposed to several days required in the case of the classic disk diffusion method. It is especially important to provide timely results if the growth of bacteria is very slow (for example, in bacteria with an acid-resistant cell wall), which requires weeks to give a recommendation on antibiotic, while the use of a sensor that detects nanomotion makes it possible to determine antibiotic resistance in *Mycobacterium* in a few hours (Mustazzolu et al., 2019). New systems based on the combination of piezoelectric elements, microfluidics, and optics allow detecting the sensitivity and/or resistance of bacteria to antibiotics in 7–15 min (Johnson et al., 2017). The use of a cantilever functionalized with antibodies can significantly reduce the time of specific diagnostics of the pathogen, since a unique immune complex is formed. At the same time, the sensitivity of the method turns out to be very high: a few hundred bacteria adhered to the cantilever are enough to obtain a reliable and reproducible signal, and this number can even drop to one single cell in the case of larger microorganisms such as yeast (Kasas et al., 2021). Thus, using only the oscillations of the AFM cantilever, it is possible to carry out a full cycle of express diagnostics includes both determining the type of microorganism and antibiotic resistance.

In general, nanomotion characterizes the metabolic activity of bacteria and can be considered as a kind of “signature of life” (Kasas et al., 2015). The binary answer of the system, i.e., presence of “nanomotion equals life” and “no nanomotion signifying death” is very convenient and seems universal. So, just as in the case of bacteria, nanomotion detection can be used to select antitumor therapy, when cancer cells are fixed on the cantilever and effective therapeutic agents are selected according to the change in the cantilever oscillations (Wu et al., 2016; Stupar et al., 2021).

In 2021, it was found that the AFM oscillation is so sensitive and specific that it can even be used to distinguish virulent from avirulent *Bordetella pertussis* cells (Villalba et al., 2021). Authors used the same virulent and avirulent *B. pertussis* Tohama I strain (avirulence was modulated by medium) with same characteristics (mass, metabolic activity, and morphology) and received different oscillation patterns depended on virulence. Thus, it is presumed that strains with more distinctions in biochemical properties, mobility and mass should have even much expressed differences in nanomotion.

In this work, we aimed (1) to compare the features of nanomotion in bacteria of different species with different biochemical profiles; (2) to detect a phagocytosis of bacteria by neutrophil granulocytes (NGs) using AFM-based nanomotion detection.

## 2. Materials and methods

### 2.1. Work with bacterial cultures

#### 2.1.1. Source of clinical isolates

The strains of microorganisms for the study were isolated on the basis of the bacteriological laboratory of Nizhny Novgorod hospital for infectious diseases No. 2: (1) *Escherichia coli* 321 was isolated from the urine of a patient with pyelonephritis, (2) *Staphylococcus aureus* 2879 M was isolated from the purulent exudate of soft tissue abscess, (3) *Proteus mirabilis* 649–2 was isolated from the feces of a patient with acute intestinal infection, and (4) *Klebsiella pneumonia* 173-P2 was isolated from the sputum of a patient with a bacterial complication after corona virus infection.

#### 2.1.2. Cultivation of bacteria

Bacteria were grown on solid fish protein hydrolysate nutrient medium (GRM) (Obolensk, Russia), sterilely transferred to the Lennox broth (LB) (Condalab, Spain), then incubated (37°C, 24 h) and washed three times with sterile PBS (450 g, 5 min). To standardize the experiments, 10 formazin turbidity units (FTU) equivalent bacteria count was used. For that, the optical density of suspensions in PBS was adjusted on the spectrophotometer Specs SSP 705 (Spectroscopic systems, Moscow, Russia) to 0.85 for *E. coli* 321, 0.74 for *S. aureus* 2879 M, 0.69 for *P. mirabilis* 649–2, and 0.73 for *K. pneumonia* 173-P2, which corresponded to the concentration of 1 × 10^9^ cells/mL used in the experiment. Bacterial morphology was analyzed using routine Gram stain.

#### 2.1.3. Biochemical profiling of bacteria

##### 2.1.3.1. Biochemical profiling of *Escherichia coli* 321, *Proteus mirabilis* 649–2, and *Klebsiella pneumonia* 173-P2

The biochemical profile of the isolates was determined according to the kit of RPC Diagnostic Systems (Nizhny Novgorod, Russia) containing specific biochemical plates for microorganism identification. The bacteria were washed off the agar with PBS (pH 6.0–6.2). 0.15 mL of the bacterial suspension was added to each well of the panel. To create anaerobic conditions in the wells for the detection of urease, lysine decarboxylase, arginine decarboxylase, arginine dehydrolase, and the formation of hydrogen sulfide, 0.1 mL of sterile petroleum jelly was added. Biochemical plates for microorganism identification were incubated (37°C, 20 h). After the end of incubation, 0.1 mL of 10% FeCl_3_ solution was added to the well to detect phenylalanine deaminase, and 0.1 mL of 40% KOH solution and 0.1 mL of α-naphthol were added to the well to determine acetylmethylcarbinol. After 3–5 h, the results were taken from the well, where β-galactosidase activity was tested, because after 20 h (standard testing time), the faint lemon-yellow color disappears in some strains.

To detect indole, 1–3 drops of Ehrlich’s reagent were added. For the detection of acetylmethylcarbinol, the results were taken 15–20 min after the addition of the reagent, since the reagent may change color.

##### Biochemical profiling of *Staphylococcus aureus* 2879 M

The bacteria were washed off the agar with 1% peptone water (pH 7.2–7.4), then a 0.15 mL of bacterial suspension was added to each well of the panel. To create anaerobic conditions, 0.1 mL of sterile petroleum jelly was added to the wells for the determination of arginine dehydrolase and urease. Biochemical plates for microorganism identification were incubated (37°C, 20 h). At the end of the incubation, 0.1 mL of 40% KOH solution and 0.1 mL of α-naphthol were added to the well to detect acetylmethylcarbinol; 0.1 mL of Griess reagent was added to determine phosphatase. Acetylmethylcarbinol result was taken 10–15 min after adding chemical agents, as the reagent may change color. The overall result of the experiment was obtained after 20 h.

#### 2.1.4. Functionalization of the cantilever and fixation of bacteria on it

Twenty microliter 0.01% solution of poly-*L*-lysine (Sigma-Aldrich, United States) was deposited onto the Si_3_N_4_ cantilever (C-MSCT, Bruker, Billerica, MA, United States) with f_0_ 4–10 kHz and k – 0.010 N/m and incubated for 10 min at 20°C. Then the nanomotion of the functionalized cantilever was recorded for control proposes. Eventually, a suspension of bacteria in LB (1 × 10^9^ cells/mL) was applied onto the cantilever, incubated for 30 min at 37°C, and fixed in the SMENA atomic force microscope (NT-MDT, Russia) holder.

#### 2.1.5. Using AFM oscillation mode to detect bacterial viability

The holder was immersed in an analytical chamber with LB, the optical system was adjusted, and the difference signal between top and bottom halves of the photodiode (DFL) changes in nA were recorded in the oscillation mode (in the control a functionalized cantilever without bacteria was used; in the experiment—with bacteria). The record of the analytical signal was carried out for 15–30 min. DFL signal was used to detect cantilever bending in AFM. Nova software (version Px.3.4.0 rev 17,188, oscillation mode) was used for recording and processing of the signal.

#### 2.1.6. Scanning electron microscopy

After the experiment, to control the presence of microorganisms, bacteria were fixed on the cantilevers with glutaraldehyde (2.5%, 20 min), the cantilevers were washed three times with distilled water, dried, and imaged by scanning electron microscopy. The electron microscopic study of the probes was carried out on a JSM-IT300 LV electron microscope (JEOL, Japan) in high vacuum mode at an accelerating voltage of 5 to 20 kV and an electron beam current of no more than 0.2 nA to minimize the effect of the beam on biological samples; the resolution was at least 12 nm. The use of a reduced accelerating voltage of up to 5 kV made it possible to better detail the surface of bacteria, with a slight loss of resolution. Studies of samples with NGs were carried out at an accelerating voltage of at least 15 kV, since at a lower voltage a noticeable deterioration in image quality was observed due to the effect of static charging on organic material. Electron microscopic images of the surface of the cantilever were obtained in low-energy secondary electrons.

### 2.2. Work with neutrophil granulocytes

#### 2.2.1. Isolation of human blood NGs

NGs were obtained from stabilized by heparin (50 IU/mL) venous blood of healthy donors on the base of Nizhny Novgorod Regional Blood Center n.a. N.Ya. Klimova. The study was approved by the Bioethics Commission of Lobachevsky State University (created on November 11, 2016, order on creation No. 497-OD), protocol No. 9 dated July 17, 2017. Blood was diluted with PBS containing 0.137 M NaCl and 0.0027 M KCl, pH 7.35 (200 g, 3 min) in a ratio of 1:1. Separation was performed on a double ficoll-trazograph gradient (*ρ* = 1.077 g/mL, ρ = 1.110 g/mL, 400 g, 40 min), then NGs were washed twice with PBS (400 g, 3 min) as described in (Pleskova et al., 2018a). Cell viability before the start of the experiments was assessed by staining the cell nuclei with propidium iodide; viability was at the level of 98–99%. NGs were used at a final concentration of 1 × 10^6^ cells/mL.

#### 2.2.2. Functionalization of the probe and fixation of NGs on the cantilever

A Si_3_N_4_ cantilever (C-MSCT, Bruker, Billerica, MA, United States) with f_0_ 4–10 kHz and k – 0.010 N/m was used for attaching the NGs. For better attachment of the cells, the cantilever was coated with poly-*L*-lysine (20 μL, 0.01%), then washed three times with a sterile normal saline solution (10 μL), and incubated (37°C, 20 min) with cell suspension (10 μL). After that, the cantilever with NGs was washed three times with PBS. The concentration of NGs was selected in a series of preliminary experiments (Pleskova et al., 2020). The prepared cantilever with cells was put on a control head.

The control signal of clear cantilever deflection was recorded as described in paragraph 2.1.5. A suspension of *S. aureus* 2879 M in HBSS (PanEco, Russia) buffered with 0.01 M HEPES (PanEco, Russia) was added into the analytical chamber at a final concentration of 5 × 10^7^ cells/mL, pipetted, and the change in the DFL signal was recorded for 30 min. For negative control NGs on the cantilever were fixed by 99.8% methanol (Sigma-Aldrich, United States) for 20 min then cantilever deflections were studied in the system with bacteria.

Immediately after the experiment the NGs on the cantilever were fixed and studied using scanning electron microscope JSM-IT300LV (JEOL, Japan). The procedures of fixation and studying described in paragraph 2.1.6.

#### 2.2.3. Study of reactive oxygen species production by the NGs population using luminol-dependent chemiluminescence

NGs were utilized no later than 120 min from the moment of blood sampling, since the production of ROS by these cells is maximum (Obraztsov et al., 2015). Cell preparation was carried out in siliconized tubes to avoid cell priming.

The isolated NGs (final concentration 1 × 10^6^ cells/mL) were incubated (15 min, 37°C) to stabilize the fluorescent activity, then mixed in a vial with bacteria (final concentration of bacteria 5 × 10^8^ cells/mL) and 100 μL of luminol (final concentration 10^−5^ M). A suspension of NGs in PBS with 100 μL of luminol served as a control.

The respiratory burst of NGs was assessed on a Lumat^3^ LB 9508 chemiluminometer (Berthold Technologies, Germany), which recorded the amount (in relative units) of free radicals formed. Values were taken discretely every 6 min for 2 h. The obtained curves were used to determine the main indicators of biochemiluminescence (BCL): the time of the peak, BCL intensity, and the integral value of the light sum.

### 2.3. Data processing and statistical analysis

The frequencies corresponding to various external signals were removed from the obtained data array using the Fourier transform (FFT). FFT was performed with the Origin 8 (OriginLab, United States). Lower bound was 2 Hz as this signal characterizes power grid fluctuations and mechanical external fluctuations such as own vibration of building. Upper bound was limited to 8 Hz as usual oscillation induced by bacteria stays around 1.6–5.1 Hz (Villalba et al., 2018). The signal was normalized according to the integral laser signal, to remove variations in reflection ability of the surface of cantilevers from different sets. While the overall signal depends on reflection ability, it needed to be processed to achieve the reproducible ratio DFL/Laser. The variance of the signal was determined after the previously mentioned filtration. Statistical processing was performed using same software. The boundaries of the normal distribution of quantitative indicators of the samples were determined using the Shapiro–Wilk test. Since the distributions were normal, the mean and standard deviation were used. The parametric Student test was used to compare two samples.

## 3. Results

### 3.1. Study of differences in the nature of nanomotions depending on the species, morphology, and biochemical profile of bacteria

**Figure 1:**
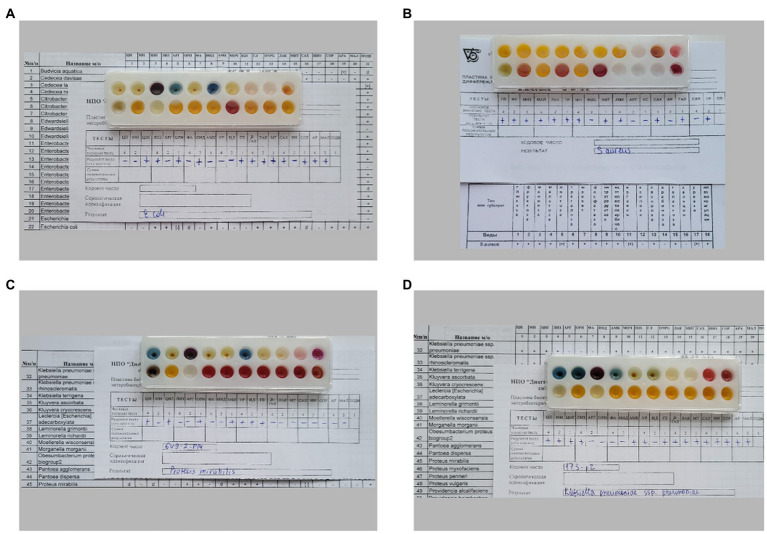
Different biochemical profiles of bacteria, determined by biochemical differentiating plates for microorganism identification: **(A)** *E. coli* 321, **(B)** *S. aureus* 2879 M, **(C)** *P. mirabilis* 649–2, **(D)** *K. pneumoniae* 173-P2.

The results of the biochemical profile study of the strains *E.coli* 321, *S. aureus* 2879 M, *P. mirabilis* 649–2, and *K. pneumonia* 173-P2 are shown in Figure 1.

**Figure 2:**
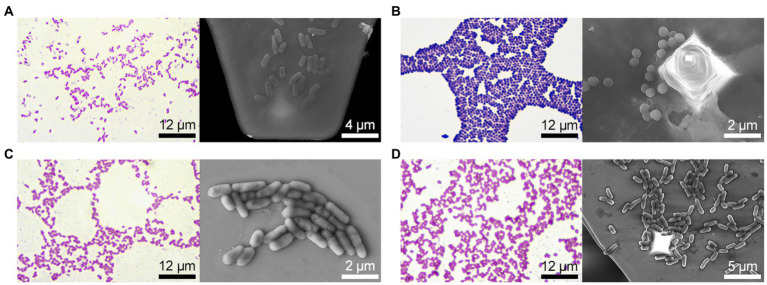
Bacteria morphology (on the left – Gram stain and optical visualization (Olympus IX71, Japan), on the right—bacteria are fixed on the cantilever immediately after the experiment on nanomotion detection, images were obtained on a scanning electron microscope): **(A)** *E. coli* 321, **(B)** *S. aureus* 2879 M, **(C)** *P. mirabilis* 649–2, **(D)** *K. pneumonia* 173-P2.

All strains fermented glucose and did not ferment dehydrogenate arginine; however, the studied bacterial strains differed significantly in other biochemical characteristics (Table 1). In particular, *E. coli* 321 was not able to form acetylmethylcarbinol, and *P. mirabilis* 649–2 was not able to utilize maltose and sucrose, while other strains could. Thus, the biochemical profile and metabolic activity of the studied strains were different. The morphology of the bacteria also varied greatly. Figure 2 shows the morphology of bacteria in Gram stain and the morphology of bacteria on the cantilever (bacteria were fixed with 2.5% glutaraldehyde immediately after the end of the nanomotion detection experiment). There are typical uniformed rod-shaped *E. coli* and globular *S. aureus* with smooth surface of cells in Figures 2A,B. However, *P. mirabilis* in Figure 2C has some structural defects of the surface. For *K. pneumoniae* (Figure 2D) the division of cells can be observed, which is proven by the cellular localization. The number of bacteria adhered to the surface of the cantilever varied between experiments due to slightly different sedimentation, adhesion properties and binning of the cantilever.

**Table 1:** Biochemical profiles of different strains.

Test	*E. coli* 321	*S. aureus* 2879 M	*P. mirabilis* 649–2	*K. pneumoniae* 173-p2
Arginine dehydrolase presence				
Ornithine decarboxylase presence				
Indole production				
Acetylmethylcarbinol				
Phenylalanine deaminase				
Urease presence				
Lysine decarboxylase presence				
H_2_S production				
Phosphatase presence				
Nitrate reductase				
Utilization of sodium citrate				
Utilization of sodium malonate				
Utilization of sodium citrate with glucose				
β-galactosidase presence				
Utilization of glucose				
Utilization of lactose				
Utilization of mannitol				
Utilization of sucrose				
Utilization of fructose				
Utilization of mannose				
Utilization of trehalose				
Utilization of inositol				
Utilization of sorbitol				
Utilization of xylose				
Utilization of galactose				
Utilization of salicin				
Utilization of arabinose				
Utilization of maltose				

████—a positive response; ████—a negative response; ████—no data.

**Figure 3:**
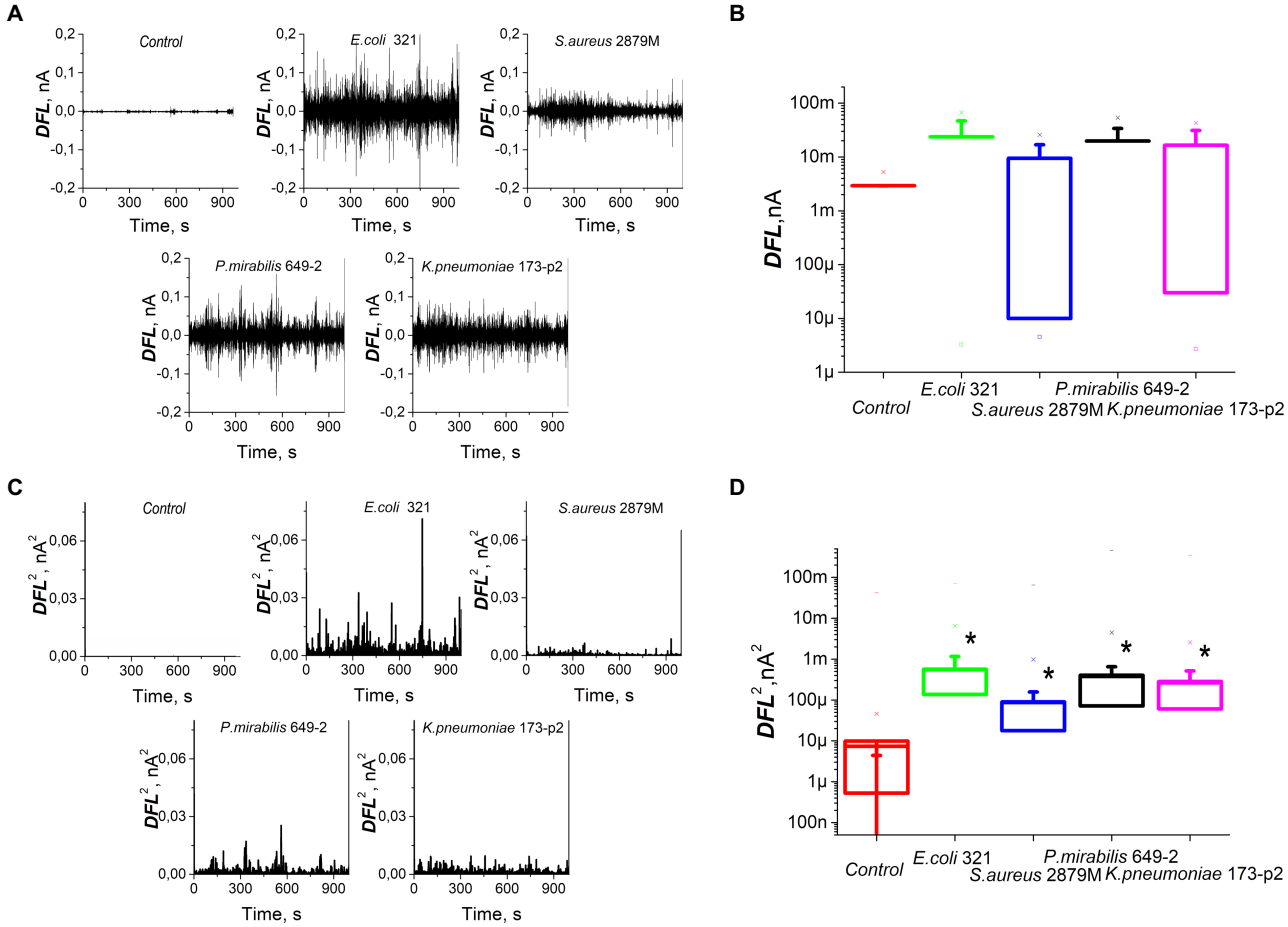
Nanomotion of different strains of bacteria on the cantilever of an atomic force microscope: **(A)** values of DFL oscillations after applying Fourier transform, **(B)** statistical processing of the DFL analytical signal, **(C)** variance of the DFL analytical signal, **(D)** variance averaged values. *—significantly different with control (*p* < 0.01).

The study of bacterial nanomotion recorded by cantilever oscillations showed that the nature of these nanomotions also varied significantly (Figure 3A). However, the average values of the DFL signal did not show statistically significant differences (*p* > 0.05, Figure 3B). In order to analyze the differences in bacterial nanomotion depending on the differences in their morphology and biochemical profile, the variance (standard deviation) was used, which showed statistically significant differences (*p* < 0.01) in the values of the frequency response during cantilever oscillations caused by metabolic activity of bacteria (Figures 3C,D). However, there were similarities in oscillations within same strain in repetitions and own ‘signature’ features of different strains (Supplementary Figures S1, S2).

### 3.2. Generation of nanomotion of NG while carrying out the physiological process of *Staphylococcus aureus* 2879 M phagocytosis

**Figure 4:**
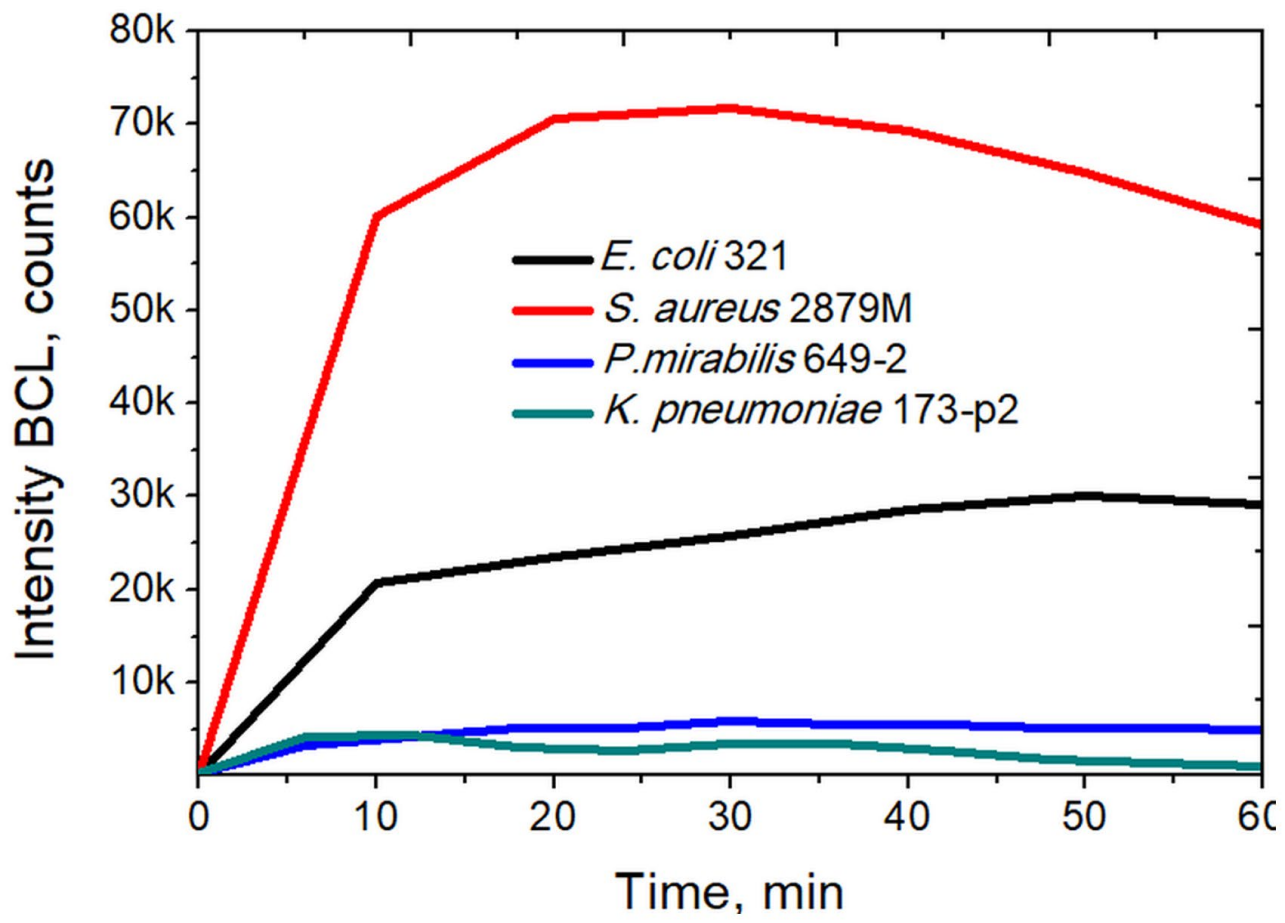
Intensity of biochemiluminescence of neutrophil granulocytes after stimulation with different strains of bacteria.

In a next step, we studied the activity of NGs during phagocytosis of *S. aureus* 2879 M bacteria. This strain was chosen since it induced the maximum production of reactive oxygen species (ROS) by NGs, as recorded in a preliminary series of experiments. These tests, based on luminol-dependent chemiluminescence (Figure 4) aimed to determine the ROS production during phagocytosis.

**Figure 5:**
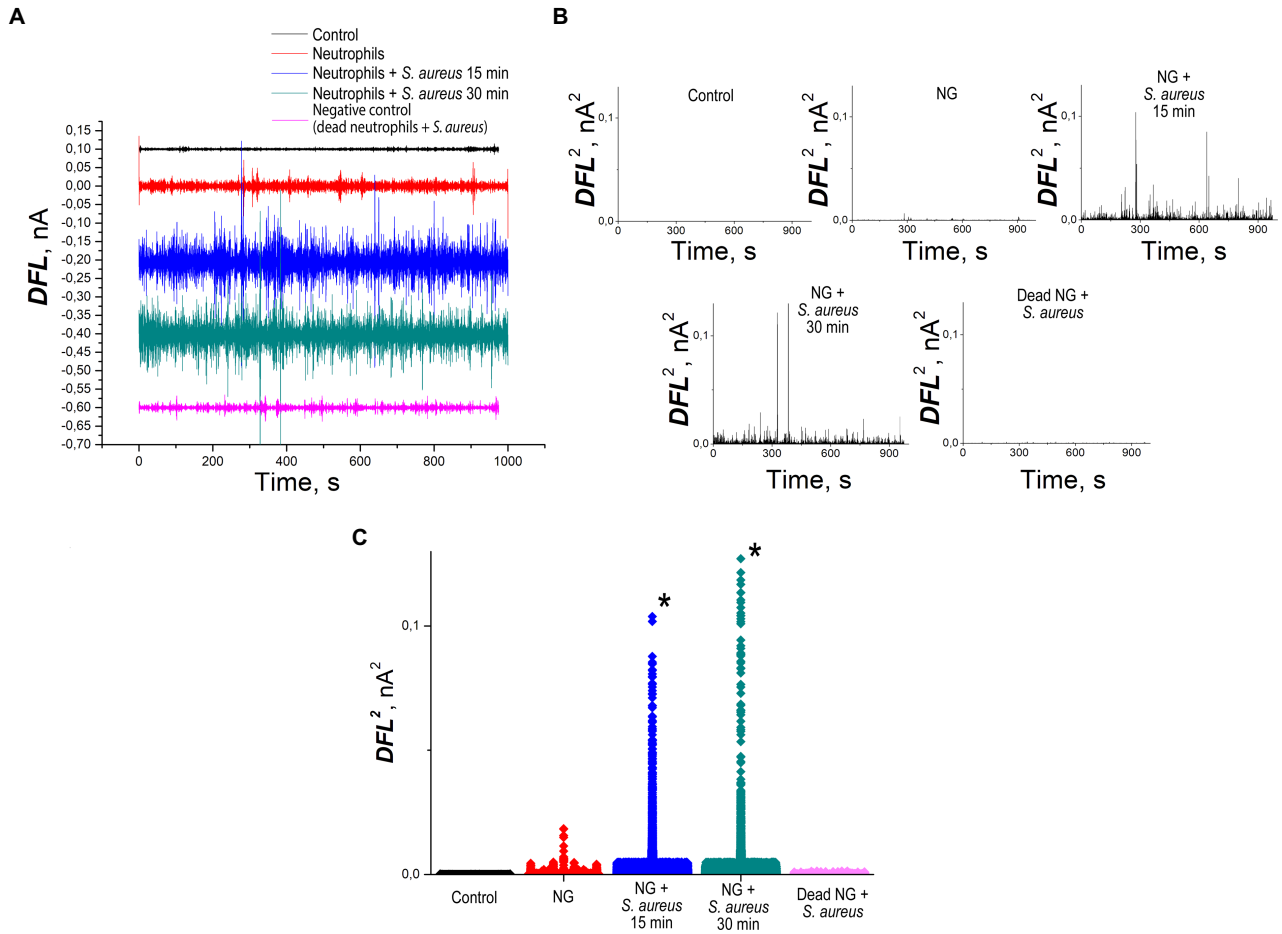
Nanomotion of NG on the cantilever of an atomic force microscope: **(A)** values of DFL oscillations after applying Fourier transform **(B)** variance of the DFL analytical signal: control—deflection of the cantilever without cells, positive control—nanomotion of NGs without stimulation, experiment—nanomotion of NGs 15 and 30 min after the insertion of the cantilever into the analytical chamber with bacteria, negative control—fluctuations of cantilever with fixed by methanol NGs, **(C)** statistical processing of the variance averaged values. *—significantly different with control (*p* < 0.01).

Cantilever oscillation in the process of *S. aureus* phagocytosis by NGs is presented in Figure 5. The values of DFL oscillations after applying Fourier transform are shown in Figure 5A. Figure 5B depicts the cantilever oscillation variance in control, unprimed NGs, NGs in the process of *S. aureus* 2879 M phagocytosis after 15 min and after 30 min and negative control of fixed NGs. The latter showed death NGs did not cause any specific oscillation like living cells, and the rest signal appeared due to insertion of the cantilever in the analytical chamber with bacteria. Figure 5C shows the variance averaged values.

**Figure 6:**
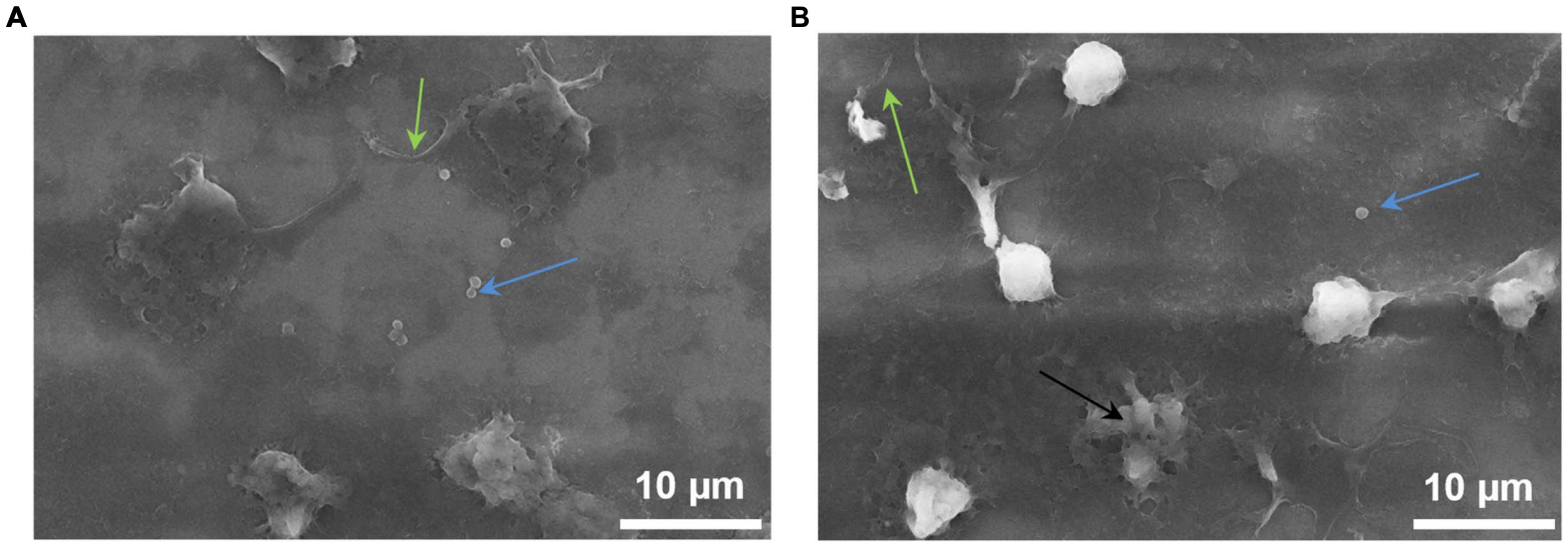
NGs fixed on the surface of the cantilever with 2.5% glutaraldehyde immediately after the end of the experiment on the detection (in the oscillation mode) of nanomotion in the process of phagocytosis. The images were taken on a JEOL (Japan) scanning electron microscope: green arrows—pseudopodia, blue arrows—*S. aureus*, black arrow—dead neutrophil.

SEM images demonstrate that NGs located on the surface of the cantilever carried out phagocytosis. It is strongly suggested by the formation of pseudopodia by cells and the presence of staphylococci inside the NGs (Figure 6).

## 4. Discussion

For these experiments, we analyzed different clinical bacterial isolates obtained from different patients with different pathologies. Only two biochemical traits of the 28 studied ones were common to all isolated strains: all of them fermented glucose and did not dehydrogenate arginine. Otherwise, the biochemical profiles of the studied bacteria were significantly different. Some significant differences were also noted in the morphology and ultrastructure of bacteria (Figure 2). The profile of bacterial nanomotion studied by AFM-based nanomotion detection also showed that each strain has its own ‘signature’ which was revealed by the change in the DFL signal (Figure 3A). Interestingly, the mean values of the DFL signal did not show statistically significant differences (*p* > 0.05, Figure 3C), so this criterion cannot be used to describe the features of nanomotion of different strains. On the contrary, variance seems to be a criterion that maximally reflects the features of bacterial nanomotion. The analysis of the distribution of variances (Figure 3C) showed that the maximum changes in the DFL signal have been noted in *E. coli 321* and *P. mirabilis* 649–2. These strains have peritrichous flagellation, which provides them with maximum mobility under physiological conditions and at the same time constitutes one of the pathogenicity factors (Manos et al., 2004; Grognot and Taute, 2021). It can be concluded that the presence of flagella makes a certain contribution to bacterial nanomotion. However, the change in the DFL cannot be explained only by the presence of flagella, because in the other two studies, strains of *S. aureus* 2879 M and *K. pneumonia* 173-P2 also exhibit nanomotion. Although these changes were smaller compare to experiments with strains with greater activity, they were statistically significantly larger (*p* < 0.01) than the control signal obtained from the oscillation of an empty functionalized cantilever.

To study the functional activity of NGs, cells were attached to the cantilever surface. Since NGs have a wide range of adhesins, they are able to independently firmly bind to any substrate (Pleskova et al., 2018b). However, in this series of experiments, weak fixation with 0.01% poly-*L*-lysine was used. The nanomotion signal of unprimed NGs was indistinguishable from the control (results not shown). Before a significant nanomotion for different types of mammalian cells in many articles was shown such as cancer cells line MCF-7 (Wu et al., 2016), sperm cells (Wu et al., 2017), and osteoblasts and neurons (Kasas et al., 2015; Kohler et al., 2019). Also the mass of mouse fibroblasts and HeLa cells was assessed (Martínez-Martín et al., 2017). However, there were either malignantly transformed cells (HeLa, MCF-7) or osteoblasts which have high metabolic activity, or cells with high mobility (sperm cells). Whereas, NGs are a special type of cells and the absence of basic activity can be explained by the features of the neutrophil life cycle. Unprimed neutrophil has a low metabolic activity level (for example, even for the synthesis of ATP molecules it uses glycolysis but not oxidative phosphorylation (Mazaki et al., 2019)) and by this the absence of the initial nanomotion of NGs is explained. After the activation of neutrophil metabolic activity of the cell rises significantly, in particular, a respiratory burst with ROS production is generated in cells, cell migration activity and biosynthesis processes are activated, and nanomotions appear. Theoretically, the adhesion process could change the basic level of NGs priming (Vogt et al., 2018), but in our experiments, it did not lead to any detectable oscillations. In order to further study the functional activity of NGs, we induced phagocytosis, which is always accompanied by a powerful functional rearrangement of cells, such as: the assembly of the NADPH oxidase complex, the release of ROS and reactive nitrogen species, the formation of hypochloric acid and hypochlorite, the activation of all intracellular enzymes, and the formation of cytokines. It can advance so powerfully that in some cases, it leads to cell death (Pleskova and Mikheeva, 2018). Therefore, given the multi-stage nature of phagocytosis, more research is needed in order to determine which stages of the process have the greatest impact on the cantilever oscillation. It must be remembered that the phagocyte, in addition to the respiratory burst, can form pseudopodia and can migrate in the direction of the chemoattractant agents. All these factors can make a significant contribution to the cantilever oscillations. Since the release of ROS strongly correlates with the activation of cells, we first determined which of the strains would cause the maximum release of ROS.

Using the method of luminol-dependent chemiluminescence, it was shown that the *S. aureus* 2879 M induces maximum production of ROS by NGs, which in 20–30 min caused the release of ROS several times higher than in the case of other strains (Figure 4). In our previous studies it was also shown that NGs absorb *S. aureus* more actively than *E. coli*, which was determined by the phagocytic number and phagocytic index (for *S. aureus*, 50% phagocytosis was observed after 7 min, and for *E. coli* after 11 min) (Pleskova et al., 2018c).

The incubation of NGs with *S. aureus* 2879 M showed a statistically significant increase in the signal 15 min after co-incubation with the bacteria. The cellular nanomotion also persisted after 30 min co-incubation (Figure 5). These results do not contradict the results of ROS formation, since Figure 4 shows that by 15 min, the respiratory burst increases exponentially, and by 30 min, it is at the maximum level. Most likely, the formation of pseudopodia (Figure 6) and, possibly, limited migration of NG can also contribute to nanomotion. The possibility of the migration process is indirectly evidenced by the presence of cellular “shadows” on the cantilever at the sites of primary cell adhesion.

The implementation of the process of phagocytosis is also evidenced by Figure 6, on which bacteria inside NG are well defined. One of the NGs obviously died as a result of the phagocytosis process.

## 5. Conclusion

Nanomotion registered using AFM cantilever seems to be a universal marker of the activity of both prokaryotic and eukaryotic cells. All bacteria, regardless of the source of isolation, features of morphology, ultrastructure, and biochemical profile, caused significant changes in the DFL signal compared to the control (an empty functionalized cantilever in the same nutrient medium). We found out that the variance values are the best metric to monitor cellular nanomotion. Nanomotion was more pronounced in flagellar forms of bacteria, although the cantilever oscillation cannot be explained by the presence of flagella alone, since metabolic processes are actively running in all bacteria, which allows bacterial cells to divide.

For NGs adhered to the surface of the cantilever in the unprimed state, no change in the analytical signal was observed. However, the implementation of NGs physiological functions such as phagocytosis, which is accompanied by cell priming and rearrangement of the metabolic activity, caused an increase in cantilever oscillation, which was observed after a 15 min co-incubation with bacteria and remained high for up to 30 min. Thus, nanomotion directly reflects the metabolically active state of cells.

A graphical abstract of study showing the affection of different bacterial and eukaryotic cells factors on the oscillations of cantilever is presented in Supplementary Figure S3.

## Data availability statement

The raw data supporting the conclusions of this article will be made available by the authors, without undue reservation.

## Ethics statement

The studies involving human participants were reviewed and approved by Bioethics Commission of Lobachevsky State University (created on November 11, 2016, order on creation No. 497-OD). The patients/participants provided their written informed consent to participate in this study.

## Author contributions

SP and RK: conceptualization. EL: experiments with bacteria. SB and EL: experiment with neutrophil granulocytes. AB: experiments with electron microscopy. EL, RK, and SP: statistical analysis. SP: project administration. SP and NB: writing the article. NB: translation and correction. All authors have read and agreed to the published version of the manuscript.

## Funding

The work has been performed in the frame of the program of scientific academic leadership ‘Priority-2030’ of Lobachevsky State University of Nizhny Novgorod and had financial support only by Russian Science Foundation, project No. 22–14-20001.

## Conflict of interest

The authors declare that the research was conducted in the absence of any commercial or financial relationships that could be construed as a potential conflict of interest.

## Publisher’s note

All claims expressed in this article are solely those of the authors and do not necessarily represent those of their affiliated organizations, or those of the publisher, the editors and the reviewers. Any product that may be evaluated in this article, or claim that may be made by its manufacturer, is not guaranteed or endorsed by the publisher.
