# Supplementary figures and images for: Differences in bacteria nanomotion profiles and neutrophil nanomotion during phagocytosis
Source: Front Microbiol. 2023 Mar 23;14:1113353. doi: 10.3389/fmicb.2023.1113353 (PMC10076590; doi:10.3389/fmicb.2023.1113353)

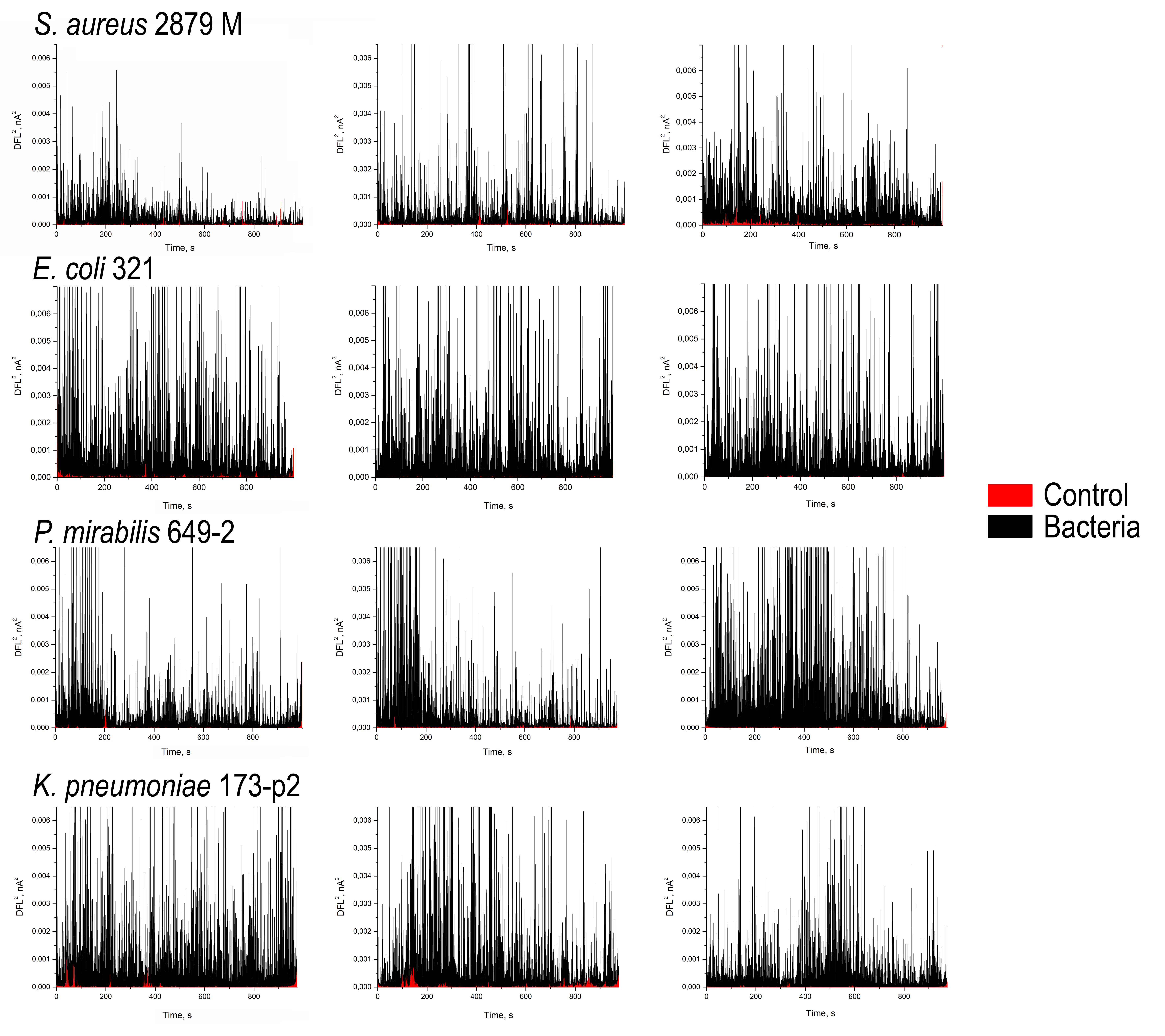

Supplement: Supplementary file 1 [file Image_1.tif]

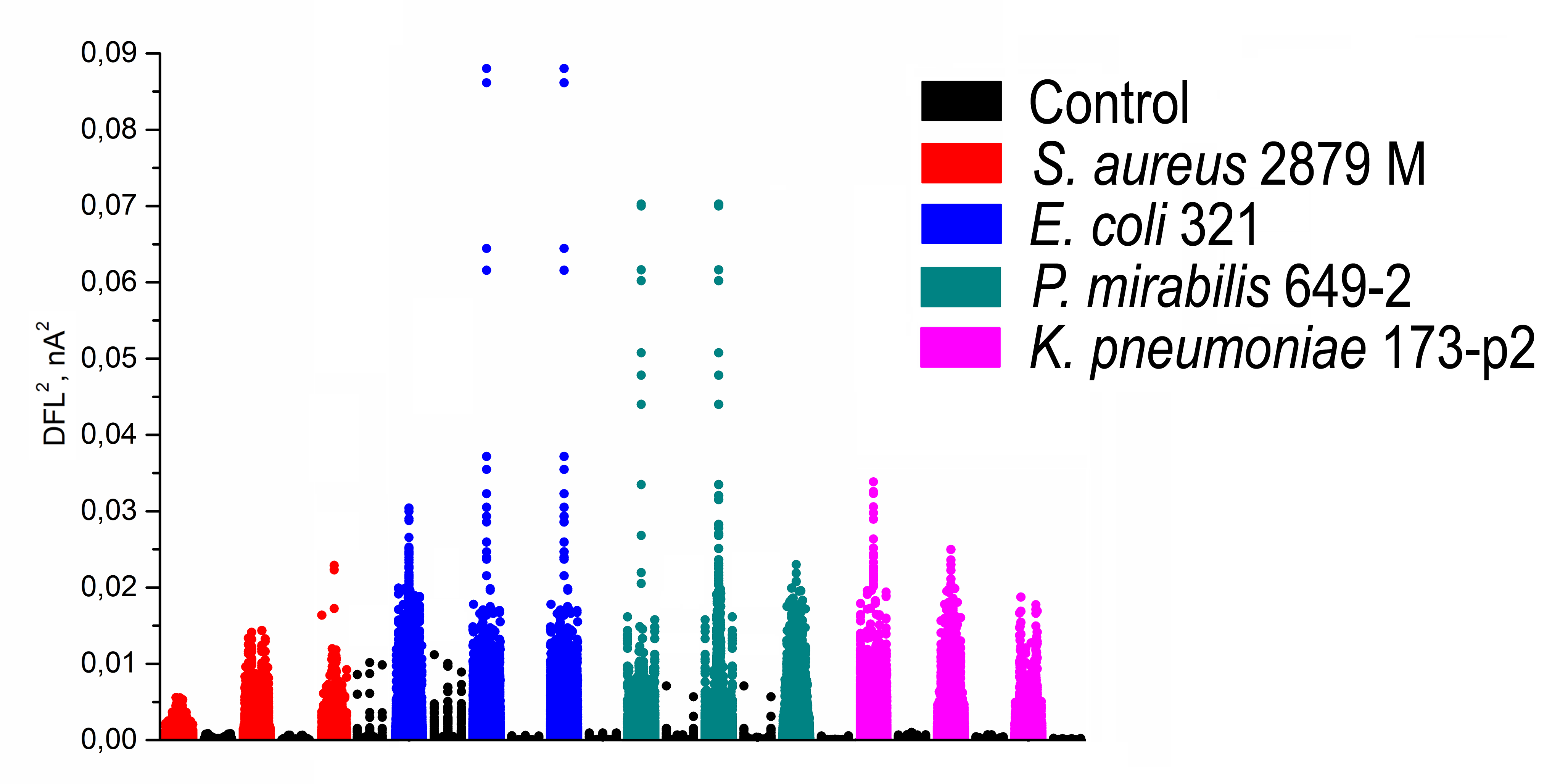

Supplement: Supplementary file 2 [file Image_2.tif]

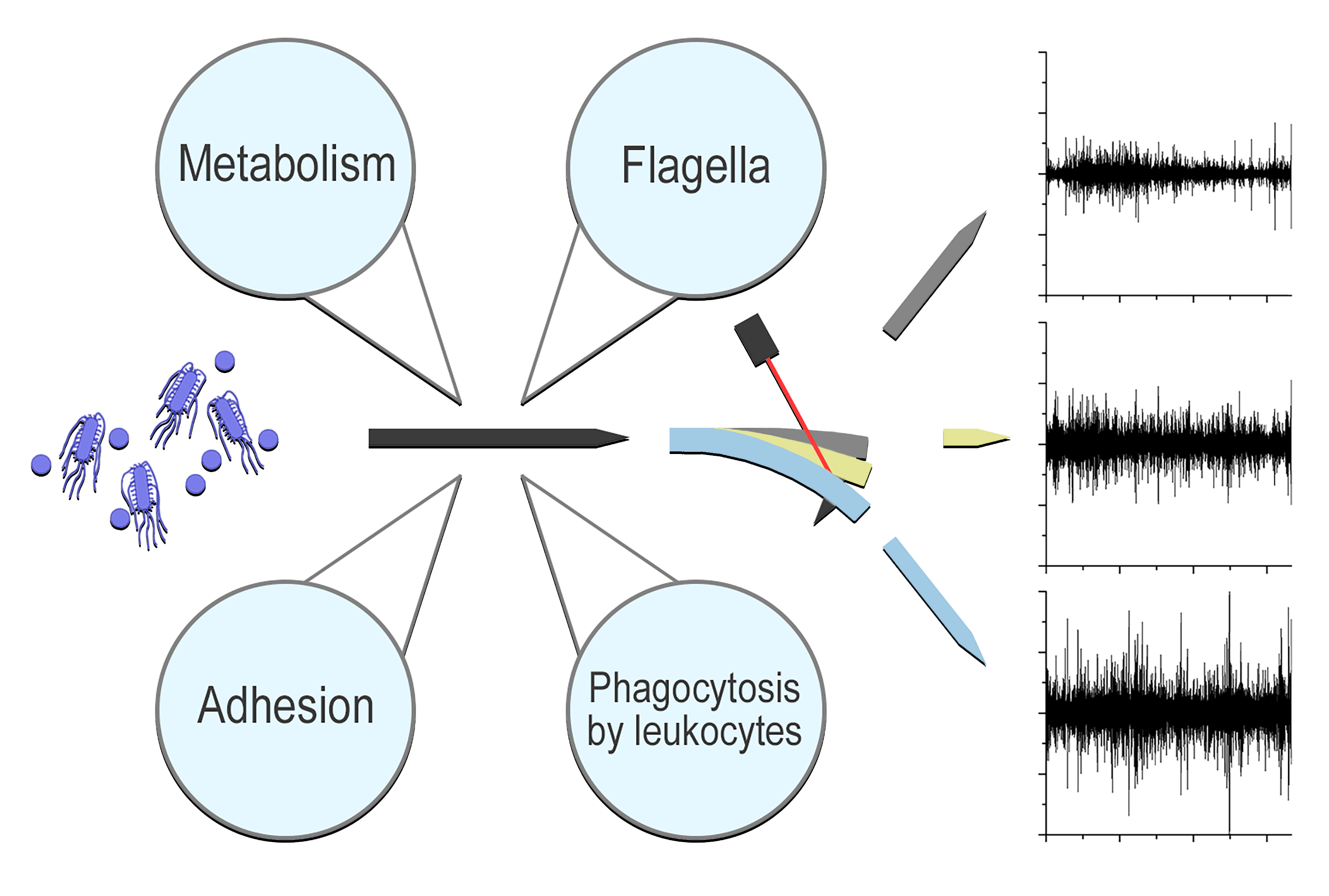

Supplement: Supplementary file 3 [file Image_3.tif]
